# Reconstruction Using a Scrotal Flap with Autologous Augmentation for Delayed Infection Caused by Penile Filler Injection: A Case Report

**DOI:** 10.3390/medicina59111998

**Published:** 2023-11-14

**Authors:** Hee-Jun Son, Woo-Sik Pae

**Affiliations:** Department of Plastic and Reconstructive Surgery, Gil Medical Center, College of Medicine, Gachon University, 24, Incheon 21565, Republic of Korea; 333jjun@naver.com

**Keywords:** penis, infections, surgical flaps, reconstructive surgical procedures

## Abstract

Penile augmentation using filler injections is gaining popularity; however, complications such as foreign body reactions can arise, leading to issues like penile ulceration and necrosis, subsequently necessitating reconstruction. The existing method of the reconstruction of the penis is primarily aimed at filling the deficit. In this paper, we describe a case in which a scrotal flap and autologous augmentation were utilized to treat a soft tissue defect caused by a delayed infection following a penile filler injection. The patient, a 41-year-old male, had received an Aquafilling^®^ (Biomedica, Prague, Czech Republic) filler injection seven years earlier and later developed a delayed infection. After debridement, the penile defect spanned the entire shaft, and the circumference of the flaccid penis was 7.5 cm. Using a bilateral scrotal flap technique, the lower margins of both flaps were rolled inward after de-epithelialization to achieve autologous augmentation. Over the three-month post-surgery follow-up, neither infections nor flap necrosis were observed. The penile circumference increased to 12 cm, and the patient reported high satisfaction with the outcome. This new surgical technique can be widely applied as treatment for a variety of penile defects.

## 1. Introduction

Many men seek to enlarge their penis size to enhance their self-esteem and improve their sexual performance [1]. Various methods can increase penile circumference, including dermal grafting from the groin, injectable fillers, prosthesis implantation, and reconstructive surgeries, with potential increases ranging from 0 to 4.9 cm [2]. Recently, non-surgical procedures have become more popular due to their reduced risks compared to surgical interventions [3]. Injectable hyaluronic acid (HA) gel is among the most prevalent soft tissue fillers used in plastic surgery [4]. The use of HA for penile augmentation is gaining traction because of its biocompatibility and minimal transient adverse effects [4].

However, penile augmentation remains a controversial topic, with no universally accepted method for this type of enhancement [5]. Complications post-penile girth augmentation using injectable fillers can vary, ranging from localized granulomas to penile abscess formation, and in some cases, diffuse cellulitis that may spread to the scrotum, perineum, or deeper pelvic organs [6].

Aquafilling^®^ (BIOTRH, spol, s.r.o., Prague, Czech Republic) fillers are reported to last between 8 to 10 years, which is more longer than HA fillers [7]. Aquafilling^®^ is a non-absorbable hydrophilic gel that was originally developed as a dermal facial filler in 2005; however, it is also used for breast and buttock augmentation [8]. The composition of Aquafilling^®^ is described as 0.9% sodium chloride solution (98% of the total volume) and cationic copolyimide (2% of the total volume) [7]. However, according to the Korean Food and Drug Administration (KFDA), its composition is 98% physiological saline and 2% polyacrylamide [9]. Numerous reports suggest that breast and soft tissue augmentation using polyacrylamide-based fillers can lead to severe side effects [10,11,12]. Consequently, Aquafilling^®^ fillers have garnered criticism and concerns regarding potential complications and are not FDA-approved for use.

Penile augmentation procedures can sometimes result in conditions like penile ulceration or necrosis, necessitating reconstructive surgery [13]. Local flaps may be advantageous for tissue reconstruction, according to the like-with-like principle [14]. However, such reconstruction may not meet the desires of patients aiming for increased penile size. We introduce a technique that utilizes bilateral scrotal flaps for both reconstruction and augmentation in penile defects arising from infections post Aquafilling^®^ filler injection.

## 2. Case Description

**Figure 1:**
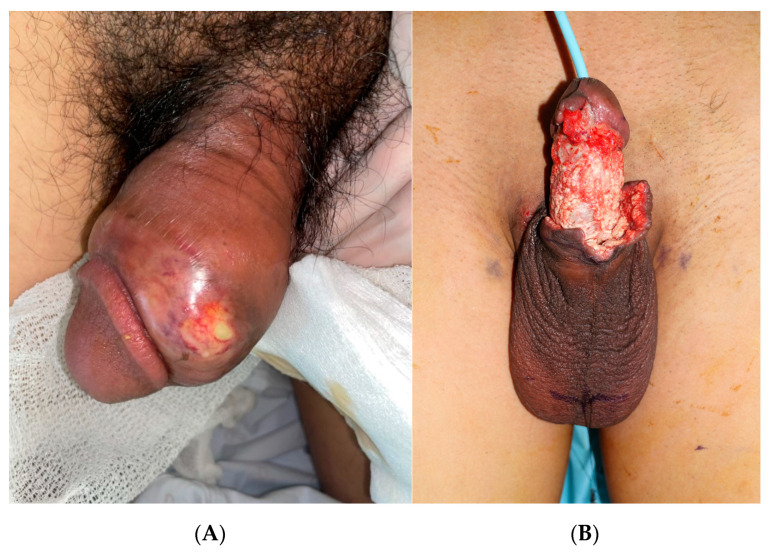
Preoperative clinical image. The penile defect was caused by a delayed infection that occurred 7 years after the filler injection. (**A**) Before and (**B**) after foreign body removal and initial debridement.

A 41-year-old man with no significant medical history underwent a penile injection of Aquafilling^®^ (Biomedica, Prague, Czech Republic) at an approved urology clinic seven years prior. He experienced no immediate complications post-procedure. However, seven years later, he presented to our hospital’s Department of Urology with pain, swelling, and purulent yellowish exudate at the injection site on his penis (Figure 1A), accompanied by a fever peaking at 38.5 °C, an indicated elevated white blood cell (WBC) count, and C-reactive protein (CRP) levels, as well as an increased erythrocyte sedimentation rate (ESR).

The Department of Urology diagnosed the patient with a delayed infection attributable to the penile filler injection. Subsequently, he underwent a foreign body removal procedure and received antibiotic treatment. A tissue culture during exploration identified Streptococcus agalactiae. This finding led to a two-week course of Trison kit inj 2 g/VI (Ceftriaxone, Sodium chloride) administered intravenously. Two weeks later, a swab culture detected methicillin-resistant Staphylococcus hemolyticus, prompting a switch to Vanco Kit inj 1 g/VI (Vancomycin, Sodium chloride 100 mL). Another week later, Pseudomonas aeruginosa was identified, resulting in a seven-day regimen of Zerbaxa inj (Ceftolozane 1000 mg, Tazobactam 500 mg), all of which were overseen by the Department of Infectious Diseases.

After incision and drainage and antibiotic treatment by the Department of Urology, skin and soft tissue defects were observed on the penis (Figure 1B). The patient was then referred to our department for reconstruction of the penile defect. Once we confirmed that no bacteria were detected at the lesion site, we planned the reconstruction using bilateral scrotal flaps with autologous tissue augmentation. Surgery was performed using a single stage. At the patient’s request, general anesthesia was administered. Fibrotic tissue was removed using Versajet (Smith and Nephews, London, UK) and Metzenbaum scissors.

After further debridement, referring to the method of Jeong [15], we designed the bilateral scrotal flap. First, the circumference and the length of the penis were measured. Both preoperative and postoperative measurements of the penis were taken in its flaccid state. The length from the tip of the glans to the penopubic junction at the dorsal side was measured, and the circumference at the base of the penis was measured. The total length of the flaccid penis was 7 cm. The circumference of the penile defect area was 7.5 cm, and the length was 5 cm.

**Figure 2:**
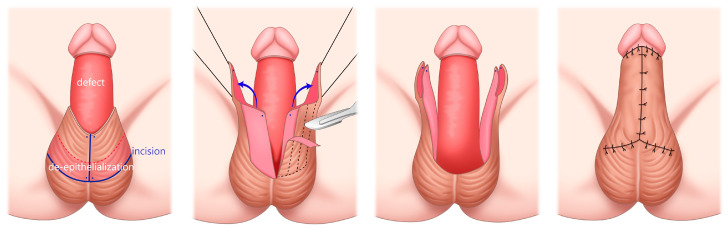
Schematic diagram of the bilateral scrotal flap with autologous augmentation. In the leftmost image, the section of the scrotum shaded in pink indicates the area designated for de-epithelialization. These areas are folded inward following the direction indicated by the blue arrow. In each flap, the locations marked by blue dots ultimately align with the anterior mid-distal region of the penile defect.

We designed an external pudendal artery-based bilateral scrotal flap to cover shaft defects referring to Lei’s method [2]. To avoid overly large flaps and minimize scrotal reduction, the flap markings were made while manually stretching the scrotum. Figure 2 offers a schematic representation of the surgical process. Unlike Lei’s technique, we doubled the vertical length of the flap, de-epithelializing half of it to achieve a penile enlargement effect. Each flap’s pedicle base was designed with a vertical span of 9 cm and a horizontal width of 5 cm, resulting in a combined horizontal width of 10 cm for both flaps. Each flap’s horizontal length matched the length of the penile defect, while its vertical length equaled the defect’s circumference. We aimed to de-epithelialize approximately half of the longitudinal length of each flap and then fold it inward. The vertical length of the scrotal flap was set to 9 cm, slightly larger than the circumference of the defect area of 7.5 cm. We planned to de-epithelialize the lower 4 cm and fold it inward and to leave the upper 5 cm exposed without de-epithelializing it.

**Figure 3:**
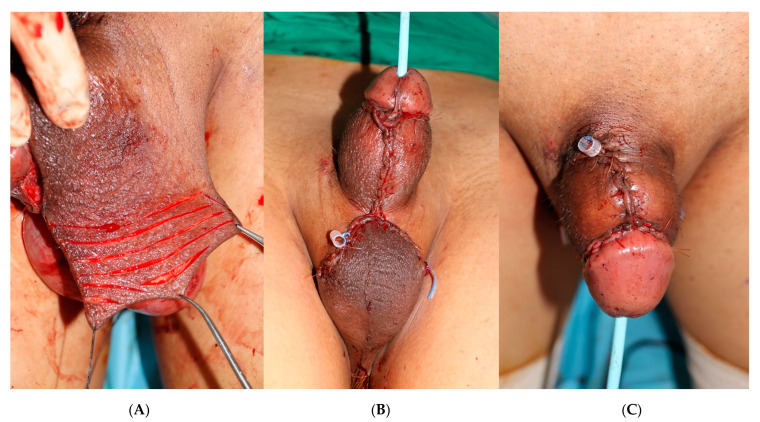
Intraoperative clinical images. The penile defect is reconstructed using an external pu-dendal artery-based bilateral scrotal flap. (**A**) De-epithelization procedure. To facilitate the process, several incisions were made prior to de-epithelialization. (**B**) The inferior view and (**C**) superior view post-surgery.

Using toothed forceps and a scalpel, we carefully separated the skin from the subdermal dartos. The use of heat energy equipment, such as a high-frequency electrotome, was strictly avoided to prevent potential damage to the blood supply of the dartos layer. The skin within the marked area was excised, revealing the donor area (scrotal dartos layer). After transpositioning both elevated flaps, the inner 4 cm edge of the bilateral flap was de-epithelialized and folded inward to achieve augmentation (Figure 2). The entire circumference of the shaft was completely covered with the bilateral scrotal flap (Figure 3B,C). Three silastic drains were placed to prevent hematoma formation. The donor site was closed primarily. The penile shaft and scrotum were dressed using fluffy gauze.

The primary endpoint was the improvement in girth at the 3-month follow-up. Postoperative penile measurements were performed in the flaccid state using the same preoperative method. Secondary outcomes include subjective satisfaction and any adverse events resulting from the procedure. Due to the absence of effective and validated measures to assess patient satisfaction with surgical intervention, satisfaction is quantitatively gauged using a visual analogue scale ranging from 0 to 10: scores from 0 to 3 are classified as very dissatisfied, 4 to 6 as dissatisfied, 7 to 8 as satisfied, and 9 to 10 as very satisfied.

**Figure 4:**
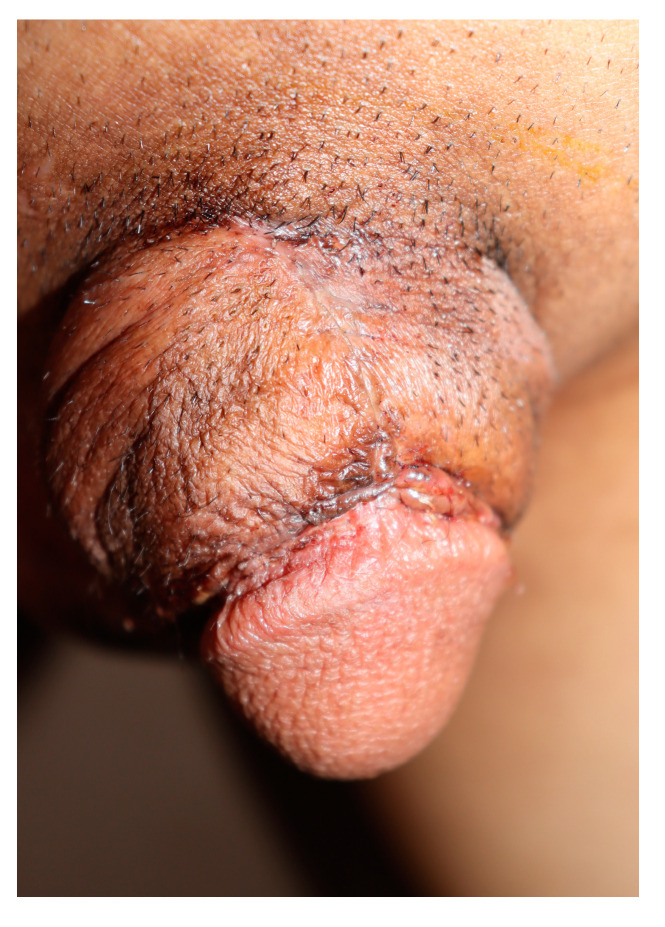
Postoperative clinical images. Two months post-surgery, the site healed without any flap necrosis or complications.

Three months post-surgery (Figure 4), the penile shaft measured 12 cm in circumference and 6.5 cm in length. Postoperatively, no skin necrosis or infections were noted at the surgical site. The patient reported a mild pulling discomfort during erection, and the postoperative sensation was diminished compared to the preoperative state. There was no postoperative pain or pressure, and no concerns regarding penile hair distribution, a potential drawback of the scrotal flap technique. The patient expressed his satisfaction level as “very satisfied”.

## 3. Discussion

Based on the range of penile dimensions for Korean adult males, the average flaccid penile length and circumference are 8.1 cm and 8.0 cm, respectively [16]. After surgery, the patient’s penile circumference expanded from 7.5 cm to 12 cm. He described his outcome as “very satisfactory”. Despite the potential for cosmetic concerns related to scrotal hair, the patient did not voice any complaints.

To our knowledge, this is the only reported case involving a bilateral scrotal flap and autologous augmentation using the de-epithelialization technique for a penile defect arising from delayed infection after an Aquafilling^®^ injection. Until recently, aesthetic outcomes of penile reconstruction were deemed secondary to functional results [17]. Various penile resurfacing methods using scrotal flaps (scrotal flap, bilateral scrotal flap, apron flap) have been documented, but most highlighted the functional impact of defect reconstruction without noting any augmentation effect [2,18].

This technique offers several advantages over existing methods [2,18]. Firstly, it enhances both functional and aesthetic outcomes, potentially boosting patient satisfaction. Secondly, our approach is versatile and applicable for various scenarios, including complications from filler injections. Penile defects might arise from multiple causes such as trauma, fasciitis, excessive circumcision, animal bites, burns, or surgery for benign or malignant lesions [19]. Thirdly, both reconstruction and enlargement can be accomplished in a single surgical stage, providing cost-effectiveness and reducing the psychological burden on patients from multiple procedures.

However, there are limitations. Firstly, patients with a smaller scrotum or a more extensive penile defect might find this method challenging due to the broad flap dimensions. Secondly, in cases where the initial size of the patient’s penis is average or above, there is a risk of overcorrection. Surgeons must ensure that patients’ enlargement expectations align with surgical plans. Operatively, surgeons must exercise caution while dissecting the dartos layer, as is the case with conventional bilateral scrotal flap techniques [2,18]. Another technical challenge lies in determining the exact widths for de-epithelialized and non-de-epithelialized areas, factoring in the flap thickness and individual scrotal stretch variances.

Our study has several limitations. Firstly, our conclusions need corroboration through larger, more extended studies. Secondly, a more prolonged follow-up is essential to identify long-term complications and functional assessments. Potential delayed surgical complications like scarring, erectile dysfunction, penile shortening, and infections should be monitored over the long term. At the 3-month post-operative mark, the patient reported minor erection-related discomfort and a slightly diminished penile sensation. Further long-term monitoring is required to understand these changes. Thirdly, the post-surgery penile length was roughly 0.5 cm shorter than pre-surgery measurements. This discrepancy might stem from measurement errors during the infection or actual post-operative shortening. The preoperative measurements were taken after the onset of infection, not before. Thus, as post-operative edema subsides, differences in circumference and length may arise, potentially leading to discrepancies.

## 4. Conclusions

This paper presented a unique case of skin necrosis caused by a delayed infection following penile augmentation using Aquafilling^®^. The defect was successfully reconstructed with a bilateral scrotal flap accompanied by autologous augmentation. Our innovative surgical technique stands out due to its ability to deliver high patient satisfaction from both functional and aesthetic perspectives. This approach offers a viable solution for various penile defects as it enables both reconstruction and cosmetic enhancement. Furthermore, with the rising trend of non-surgical penis enlargement procedures [3,4], it is imperative to promptly identify and manage infections associated with penile fillers.

## Data Availability

Data are contained within the article.
